# The Census of 1890

**Published:** 1895-03

**Authors:** 


### Editorial, Etc.

THE CENSUS OF 1890.—January, 1881 this Journal published the correspondence between Dr. T. B. Gunning and the Census Bureau which in 1880 demanded statistics on the ground that dental offices are establishments of productive industry. A similar unwarranted demand for statistics of manufactures was made of dental practitioners in 1890. While as early as January 3, 1881, Gen. Francis A. Walker, Superintendent of the Census of 1880, determined to omit from the tabulation, "Dentistry in all its branches," it was not till June 27, 1892 that Supt. R. B. Porter of the Census of 1890 agreed in writing "with the representatives of the Dental Association present at a meeting of the Committee on the Census to carry out the spirit and purposes" of an amendment to a Bill, which had passed the House of Representatives, providing penalties for refusal to answer questions included in the schedule of the Census office, that nothing in the Act "shall be construed as authority to collect statistics from professional men such as lawyers, physicians and dentists." With a similar experience it will be 1904 before the dental profession will have adjusted the case with the Census Bureau of 1900!

The American Dental Association has appointed a committee consisting of Drs. Alonzo Boice, Wm. Shaw Twilley, C. A. Meeker to draft "a sketch of the history of dentists being classified as manufacturers by the Census Bureau of 1890." and the letter published elsewhere in this Journal by one of the editors was prepared at the special request of the chairman, Dr. Boice. So far as known, no other documentary evidence has been published except the correspondence in the "Annals" of the Philadelphia County Dental Society after October 20, 1890 and the report of Dr. J. B. Rich, chairman, in the "Transactions" of the American Dental Association for 1892.

What the profession understands by a *mechanical* dentist is well expressed in a case presented to the National Association of Dental Examiners at its meeting in 1884 by Dr. E. P. Keech, President, from Maryland: "The party is a first-class mechanical workman; he has no sign, and the speaker was not sure that he had ever filled a tooth; he works in a laboratory, doing that class of work; his business was to make a pivot-tooth or a set of teeth—whatever may be given him to do." The Census office, however, had these views: (1) September, 1890, "Referring to mechanical dentists you are informed that the term is considered by this office to embrace all branches of the profession except the extraction of teeth and should be duly reported on General Schedule No. 3;" (2) October 1890, "The term "mechanical dentistry" is intended to mean that branch of the profession which includes the manufacture of artificial dentures, such as plates, crowns, caps, etc., which are manufactured wholly or in part outside the mouth, and afterwards inserted;" (3) "Regarding the production of artists in the dental trade" * * * "These men are certainly manufacturers in the same sense that a blacksmith who makes a shoe from a bar of iron and fits it to the horse's hoof is a manufacturer."

A fit conclusion is the query in an editorial in the Philadelphia *Press* on the classification of dentists, "why the repair of teeth should come under mechanical employment and the repair of the body belong to a liberal profession." R. G.

